# Supplementary material for: Dietary Patterns and Associations with Myopia in Chinese Children
Source: Nutrients. 2023 Apr 18;15(8):1946. doi: 10.3390/nu15081946 (PMC10142016; doi:10.3390/nu15081946)
Supplement: Supplementary file 1 [file nutrients-15-01946-s001.zip › Table S1. General characteristics of participants according to quartiles of the dietary patterns.pdf]

Table S1. General characteristics of participants according to quartiles of the dietary patterns

	A dietary pattern scores					B dietary pattern scores					C dietary pattern scores				
**Parameters**	**Q1**	**Q2**	**Q3**	**Q4**	***P* for trend**	**Q1**	**Q2**	**Q3**	**Q4**	***P* for trend**	**Q1**	**Q2**	**Q3**	**Q4**	***P* for trend**
Number (n)	1858	1857	1859	1857		1857	1858	1858	1858		1858	1858	1858	1857	
Sex (%)					0.565					0.302					0.510
Boy	939(50.5)	919(49.5)	902(48.5)	963(51.9)		904(48.7)	932(50.2)	958(51.6)	929(50.0)		919(49.5)	980(52.7)	900(48.4)	924(49.8)	
Girl	919(49.5)	938(50.5)	957(51.5)	894(48.1)		953(51.3)	926(49.8)	900(48.4)	929(50.0)		939(50.5)	878(47.3)	958(51.6)	933(50.2)	
Age (%)					0.182					0.001*					0.490
10	899(48.4)	937(50.5)	906(48.7)	952(51.3)		970(52.2)	930(50.1)	928(49.9)	866(46.6)		906(48.8)	959(51.6)	939(50.5)	890(47.9)	
11	959(51.6)	920(49.5)	953(51.3)	905(48.7)		887(47.8)	928(49.9)	930(50.1)	992(53.4)		952(51.2)	899(48.4)	919(49.5)	967(52.1)	
Residency (%)					<0.001*					0.007*					0.162
Urban	537(28.9)	641(34.6)	886(47.7)	1070(57.6)		816(43.9)	700(37.7)	724(39.0)	894(48.2)		776(41.8)	711(38.3)	884(47.6)	763(41.1)	
Rural	1320(71.1)	1214(65.4)	973(52.3)	787(42.4)		1041(56.1)	1151(62.3)	1134(61.0)	962(51.8)		1080(58.2)	1147(61.7)	973(52.4)	1094(58.9)	
Region (%)					<0.001*					<0.001*					0.007*
Northern China	1084(58.4)	1101(59.4)	830(44.6)	758(40.8)		823(44.3)	958(51.6)	1043(56.1)	949(51.1)		856(46.1)	1019(54.8)	924(49.8)	974(52.5)	

Southern China	773(41.6)	754(40.6)	1029(55.4)	1099(59.2)		1034(55.7)	899(48.4)	815(43.9)	907(48.9)		1000(53.9)	839(54.2)	933(50.2)	883(47.5)	
Nutrition status (%)					0.022*					0.825					<0.001*
Stunting and wasting	126(6.9)	117(6.4)	124(6.8)	132(7.2)		120(6.6)	132(7.2)	123(6.7)	124(6.8)		168(9.2)	114(6.2)	132(7.2)	85(4.6)	
Normal	1217(66.6)	1163(63.7)	1115(60.8)	1133(62.2)		1136(62.1)	1195(65.3)	1144(62.7)	1153(63.2)		1188(65.1)	1180(64.5)	1162(63.7)	1098(59.9)	
Overweight and obesity	484(26.5)	546(29.9)	595(32.4)	557(30.6)		573(31.3)	504(27.5)	559(30.6)	546(30.0)		468(25.7)	535(29.3)	530(29.1)	649(35.4)	
Moderate-to high-intensity physical activity (d/w)					0.114					0.036*					<0.001*
0	627(33.8)	593(32.0)	573(30.9)	603(32.5)		586(31.6)	613(33.0)	580(31.3)	617(33.2)		494(26.6)	593(32.0)	690(37.2)	619(33.3)	
1	273(14.7)	355(19.1)	364(19.6)	311(16.7)		322(17.3)	326(17.6)	328(17.7)	327(17.6)		316(17.0)	333(18.0)	347(18.7)	307(16.5)	
2	362(19.5)	458(24.7)	550(29.6)	461(24.8)		424(22.8)	425(22.9)	488(26.3)	494(26.6)		534(28.8)	458(24.7)	421(22.7)	418(22.5)	
≥3	595(32.0)	450 (24.2)	368(19.8)	482(26.0)		524(28.2)	492(26.5)	459(24.7)	420(22.6)		512(27.6)	471(25.4)	399(21.5)	513(27.6)	
screen time (min/d)					0.010*					0.001*					0.670
0	1(0.1)	4(0.2)	4(0.2)	6(0.3)		7(0.4)	1(0.1)	2(0.1)	5(0.3)		2(0.1)	0(0.0)	0(0.0)	13(0.7)	

>0～<60	1277(68.7)	1288(69.4)	1287(69.3)	1343(72.3)		1359(73.3)	1254(67.5)	1347(72.6)	1235(66.5)		1261(68.0)	1358(73.1)	1344(72.3)	1232(66.4)	
≥60	580(31.2)	563(30.4)	565(30.4)	508(27.4)		489(26.4)	603(32.5)	507(27.3)	617(33.2)		592(31.9)	499(26.9)	514(27.7)	611(32.9)	
Sleep time(h/w)					0.213					0.929					<0.001*
≤9	580(31.2)	531(28.6)	639(34.4)	581(31.3)		516(27.8)	675(36.3)	602(32.4)	538(29.0)		710(38.2	555(29.9)	545(29.3)	521(28.1)	
>9	1278(68.8)	1326(71.4)	1220(65.6)	1276(68.7)		1341(72.2)	1183(63.7)	1256(67.6)	1350(71.0)		1148(61.8	1303(70.1)	1313(70.7)	1336(71.9)	

P for trend was based on a linear trend test across the quartiles of dietary pattern scores (Q1–Q4), * *P* < 0.05.
